# High yield cyclotron production of a novel ^133/135^La theranostic pair for nuclear medicine

**DOI:** 10.1038/s41598-020-79198-x

**Published:** 2020-12-17

**Authors:** Bryce J. B. Nelson, John Wilson, Jan D. Andersson, Frank Wuest

**Affiliations:** 1 Department of Oncology, University of Alberta, 11560 University Avenue, Edmonton, AB T6G 1Z2 Canada; 2 Edmonton Radiopharmaceutical Center, Alberta Health Services, 11560 University Ave, Edmonton, AB T6G 1Z2 Canada

**Keywords:** Oncology, Chemistry, Materials science

## Abstract

This study reports the high-yield production of a novel ^133/135^La theranostic pair at a 22 MeV proton beam energy as an attractive alternative to the recently introduced ^132/135^La pair, demonstrating over an order of magnitude production increase of ^133/135^La (231 ± 8 MBq ^133^La and 166 ± 5 MBq ^135^La at End of Bombardment (EOB)) compared to 11.9 MeV production of ^132/135^La (0.82 ± 0.06 MBq ^132^La and 19.0 ± 1.2 MBq ^135^La) for 500 µA·min irradiations. A new sealed solid cyclotron target is introduced, which is fast to assemble, easy to handle, storable, and contains reusable components. Radiolabeling with macrocyclic chelators DOTA and macropa achieved full incorporation, with respective apparent ^133^La molar activites of 33 ± 5 GBq/µmol and 30 ± 4 GBq/µmol. PET centers with access to a 22 MeV capable cyclotron could produce clinically-relevant doses of ^133/135^La, via ^nat^Ba irradiation, as a standalone theranostic agent for PET imaging and Auger electron therapy. With lower positron energies and less energetic and abundant gamma rays than ^68^Ga, ^44^Sc and ^132^La, ^133^La appears to be an attractive radiometal candidate for PET applications requiring a higher scanning resolution, a relatively long isotopic half-life, ease of handling, and a low patient dose.

## Introduction

Theranostics in nuclear medicine is a technique whereby a site specific pharmaceutical is radiolabelled first with a radioisotope for diagnostic imaging. After analysis, the same pharmaceutical is labelled with a particle emitting radioisotope for therapeutic application^1^. The complementary isotopes used are called theranostic pairs. It is essential that the two isotopes have very similar chemical properties with the ideal case being that they are different isotopes of the same element. Auger electron-emitting isotopes have potential as a high linear energy transfer (LET) therapeutic agent to destroy cancer cells by depositing their ionizing emission energy over a very short path length, damaging DNA by inducing various types of DNA damage, including double-strand breaks. This holds advantages over lower LET therapy such as β^-^ therapy where emissions can travel over 1 cm, and may unnecessarily irradiate healthy tissue^2, 3^. High LET Auger electron emissions have achieved encouraging clinical results, with ^111^In-DTPA-octreotide and ^125^I-IUdR causing tumor remissions in patients with lower normal tissue toxicity, and improvements in the survival of glioblastoma patients using ^125^I-mAb 425 with minimal normal tissue toxicity^4^. A recently developed theranostic pair is ^132/135^La, where positron emissions from ^132^La are used for PET imaging while the Auger electrons from ^135^La have the potential for use in Auger electron therapy (AET)^5–7^. Theranostic La pairs are not only inherently useful but also can serve as surrogates for potential future study relating to ^225^Ac alpha-particle therapy. ^225^Ac-labeled compounds have seen significant recent clinical successes in treating aggressive tumor metastases^2, 8^.

However, ^132/135^La has limitations for PET imaging due to its fundamental positron and gamma emission properties, and current cyclotron production methods. The average and maximum ^132^La positron energies of 1.29 MeV and 3.67 MeV are significantly higher than those of other commonly used PET isotopes such as ^18^F (E_mean_ = 0.250 MeV, E_max_ = 0.634 MeV), ^64^Cu (E_mean_ = 0.278 MeV, E_max_ = 0.653 MeV), ^68^Ga (E_mean_ = 0.829 MeV, E_max_ = 1.90 MeV), or ^44^Sc (E_mean_ = 0.632 MeV, E_max_ = 1.47 MeV)^9^. The higher positron energy of ^132^La implies reduced PET image spatial resolution for tumor imaging, especially when imaging smaller tumors and metastases. Furthermore, ^132^La emits high abundance gamma rays within typical 511 keV PET scanner energy windows that can contribute to spurious coincidences, as well as high energy gamma rays that may complicate handling.

Using ^nat^Ba target material, current ^132^La cyclotron production methods via ^132^Ba(p,n)^132^La require long irradiation times and generate reduced activity due to the very low natural abundance of ^132^Ba (0.1%).

The present work describes high yield ^133/135^La production through 22 MeV proton irradiation of ^nat^Ba metal encapsulated within a convenient sealed cyclotron target. Irradiating ^nat^Ba at 22 MeV generates much higher yields of ^133/135^La compared to ^132/135^La production at 11.9 MeV and bypasses the majority of ^132^La production, avoiding contributions from its higher energy positron emissions. ^133^La has average and maximum positron energies of 0.461 MeV and 1.02 MeV, respectively, that are lower than those of ^132^La and other PET isotopes such as ^68^Ga and ^44^Sc. Gamma emissions from ^133^La are low intensity and energy, falling well outside the typical PET scanner energy window. These features of ^133^La simplify handling and reduce patient dose. This novel ^133/135^La isotope system and its production method have the potential to improve the image quality of smaller and metastatic tumors and allow clinically relevant production of ^133/135^La via shorter cyclotron beamtime irradiations without requiring isotopically enriched Ba target material. High-yield production is possible via proton irradiation of ^nat^Ba on a cyclotron capable of attaining 22 MeV beam energies. The favorable ^133^La positron and gamma-ray emission properties suggest that ^133/135^La has significant potential as a theranostic pair substitute for ^132/135^La.

## Materials and methods

### Chemicals

Natural barium (99.99% trace metals basis) dendritic pieces, ACS reagent grade concentrated hydrochloric acid (37%) and nitric acid (70%), and ICP-OES elemental standards were purchased from Sigma-Aldrich (St. Louis, MO, USA). Silver rod (99.9%) was purchased from Metal Supermarkets (Mississauga, ON, Canada). Branched DGA resin (50–100 µm) was purchased from Eichrom (Lisle, IL, USA). NIST traceable γ-ray sources used for high-purity germanium detector (HPGe) energy and efficiency calibration were acquired from Eckert & Ziegler Isotopes (Valencia, California, USA). Thin-layer chromatography silica gel sheets were purchased from Merck (Darmstadt, HE, Germany).

High purity water (18 MΩ·cm) was obtained from a MilliporeSigma Direct-Q 3 UV system (Burlington, MA, USA). The macrocyclic chelator DOTA was purchased from Macrocyclics (Plano, TX, USA), and the macrocyclic chelator macropa was purchased from MedChemExpress (Monmouth Junction, NJ, USA).

### Instrumentation

Sample activity was measured using an Atomlab 500 Dose Calibrator (Biodex, Shirley, NY, USA). Radionuclidic purity was assessed using a GEM35P4-70-SMP high-purity germanium detector (ORTEC, Oak Ridge, TN, USA) with ORTEC GammaVision software. Elemental purity was assessed using a 720 Series ICP-OES (Agilent Technologies, Santa Clara, CA, USA). A NEPTIS Mosaic-LC synthesis unit (Optimized Radiochemical Applications, Belgium) was used to separate and purify the ^133/135^La from the dissolved Ba target solution. An AR-2000 Radio-TLC Imaging Scanner (Eckert & Ziegler, Hopkinton, MA, USA) was employed to quantify the fraction of chelator-bound ^133/135^La after the reaction. The solid targets were manufactured using a Model 6318 hydraulic press (Carver, Wabash, IN, USA), and the ^nat^Ba metal was pressed inside a 10 mm (I.D.) EQ-Die-10D-B hardened steel die (MTI Corporation, Richmond, CA, USA). A S90013A optical light microscope (Fisher Scientific, Waltham, MA, USA) was employed to inspect the seal integrity of each sealed solid target after manufacturing.

### Cyclotron targetry and irradiation

**Figure 1:**
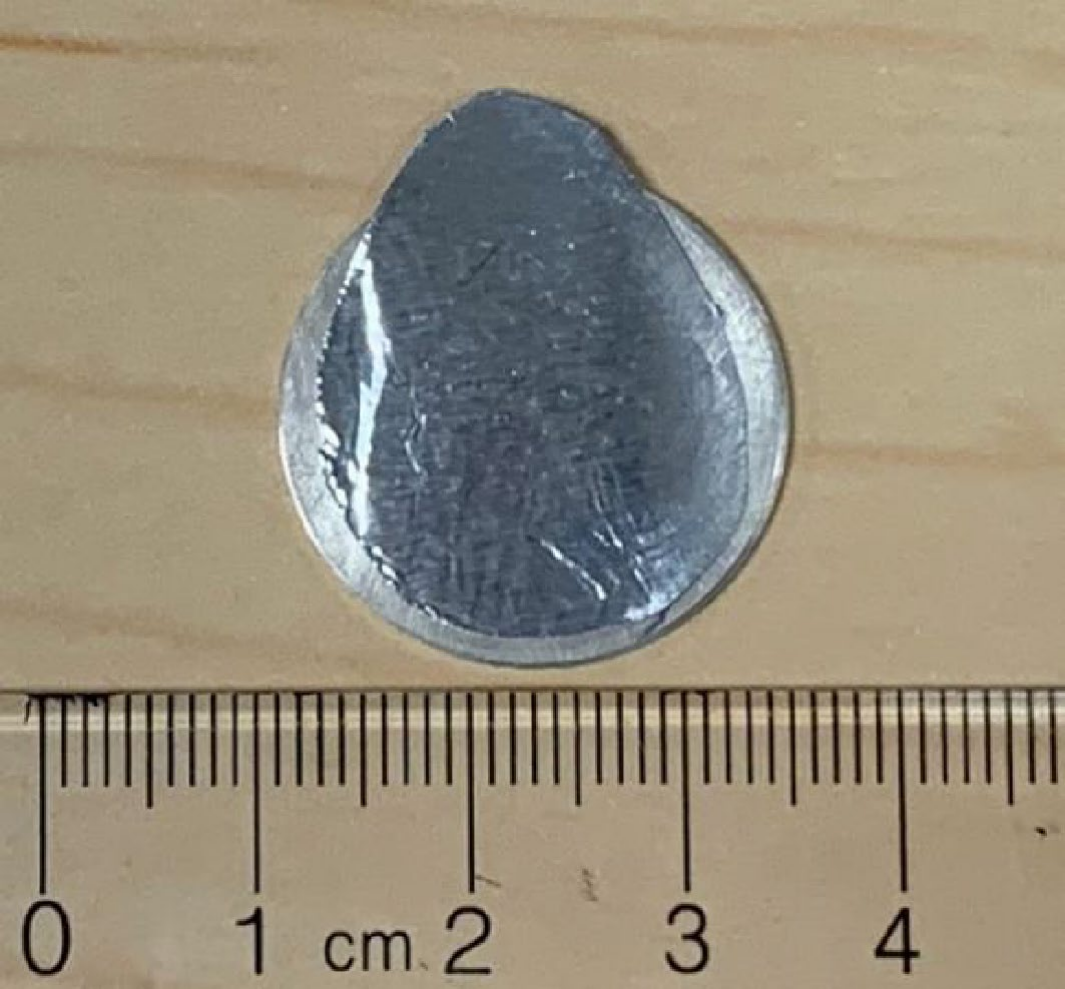
A completed ^nat^Ba sealed cyclotron target ready for irradiation.

A completed sealed cyclotron target is depicted in Fig. 1. Cyclotron targets were prepared from 200 mg of ^nat^Ba metal, an Ag disc (24 mm diameter, 1.5 mm thick) cut from an Ag rod, In wire (1 mm diameter), and Al foil (25 µm thick). A 10 mm diameter depression was machined into the center of each disc to a 100 µm depth, and a 1 mm wide annulus with an inner diameter of 15 mm was machined to a depth of 100 µm. Using a method similar to the target production described in^10^, ^nat^Ba metal was quickly loaded into a hardened stainless steel die to minimize exposure to the atmosphere, and a force of 15 kN was applied using a hydraulic press, producing a 10 mm diameter pellet with a thickness of 0.8 mm. Pellets were produced in large quantities (> 10/batch) and removed quickly from the die and sealed in a vial with an argon atmosphere to prevent oxidation during storage.

A 23 mm diameter Al foil cover was cut out with a flap extension to facilitate post-irradiation removal by peeling. Individual pellets were then placed in the central Ag disc depression and pressed at a force of 20 kN on the hydraulic press to secure the pellets in the depression. 5.5 cm of In wire was then laid into the annulus depression with 1 mm of overlap at the ends, the target assembly was quickly covered by the Al cover, and a force of 25 kN was applied using the hydraulic press to compress the In wire to form an air-tight bond between the Ag disc and Al cover. Following pressing, the target was observed under an optical light microscope to confirm target seal integrity, verifying there were no pinholes present in the Al cover. The target was stored under regular atmospheric conditions ready for on-demand irradiation.

Targets were irradiated at 22 MeV using a 24 MeV TR-24 cyclotron (Advanced Cyclotron Systems Inc., Richmond B.C., Canada) for 25–200 min with a maximum proton beam current of 20 µA at current densities of 25.5 µA/cm^2^. A pneumatically actuated TA-1186 solid target assembly (Advanced Cyclotron Systems Inc., Richmond B.C., Canada) was used with the target disc perpendicular to the proton beam. O-rings within the assembly provided a helium gas seal on the front and water seal on the back for both cooling streams. The Ag target was designed to be at least 0.6 mm thick behind the 0.8 mm thick ^nat^Ba pellet so that the exit beam energy leaving the Ag disc was degraded below 6 MeV, as simulated by SRIM 2013^11^. This design consideration was to avoid the production of ^13^N (t_1/2_ = 9.97 min) in the cyclotron cooling water circuit via the ^16^O(p,α)^13^N reaction. A 250 µm thick Ag degrader was added to the cyclotron beamline after the Al vacuum foil so that extracting the cyclotron beam at 17 MeV resulted in the target incident energy being degraded to 11.9 MeV. These irradiations at 11.9 MeV served to provide a comparison to the ^132/135^La isotope production introduced by Aluicio-Sarduy et al.^5^.

After allowing 1–2 h post-irradiation for decay of short-lived La isotopes, the target assembly was opened pneumatically, and the sealed target slid down a plastic guide tube into a lead shield. The lead shield was brought to a dose calibrator where its activity was measured, followed by placement into a lead castle containing a NEPTIS automated separation unit.

### Nuclear reaction cross-sections of interest

**Figure 2:**
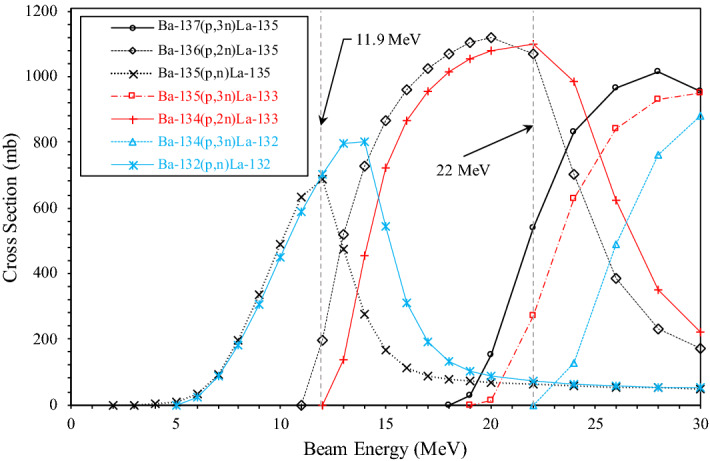
Nuclear reaction cross-section simulation data of the proton-induced nuclear reaction on ^132/134/135/136/137^Ba for ^132/133/135^La^12^.

**Figure 3:**
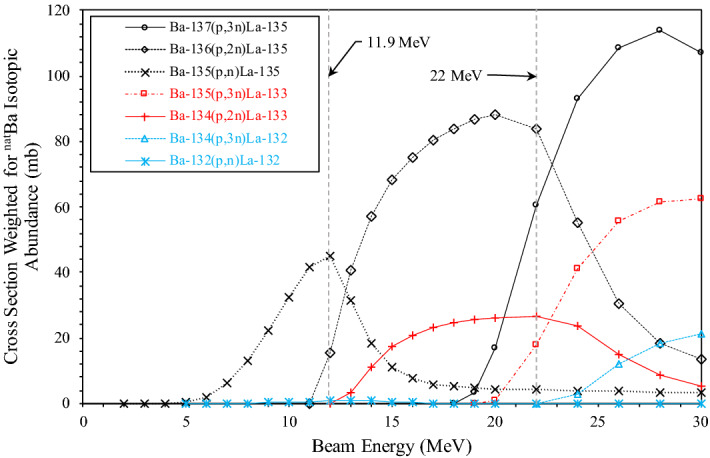
Nuclear reaction cross-section simulation data of the proton-induced nuclear reaction on ^132/134/135/136/137^Ba for ^132/133/135^La weighted for ^nat^Ba isotopic abundance^12^.

Nuclear reaction cross-sections simulated by TENDL 2019 for the ^13x^Ba(p,xn)^13x^La reactions of interest for ^132/133/135^La are shown in Fig. 2. These same cross-sections, weighted for ^nat^Ba isotopic abundance, are displayed in Fig. 3. The cyclotron beam was extracted at an energy of 22.2 MeV and degraded to a target incident energy on ^nat^Ba of 22 MeV. The target incident energy of 22 MeV was selected using TENDL 2019 cross-section simulation data^12^.

At a 22 MeV target incident beam energy, the simulation suggests significant ^135^La and ^133^La cross sections for the ^137^Ba(p,3n)^135^La, ^136^Ba(p,2n)^135^La, ^135^Ba(p,3n)^133^La, and ^134^Ba(p,2n)^133^La reactions. The ^132^Ba(p,n)^132^La cross-section is over two orders of magnitude lower at 22 MeV compared to at 11.9 MeV, and the ^134^Ba(p,3n)^132^La reaction cross-section does not begin until just above 22 MeV. Irradiating ^nat^Ba at 22 MeV should therefore maximize the production of ^133^La and ^135^La, bypass the majority of ^132^La production from the ^132^Ba(p,n)^132^La reaction, and just avoid the onset of the significant ^134^Ba(p,3n)^132^La reaction. Due to the higher natural abundances of ^134^Ba (2.42%) and ^135^Ba (7.59%) compared to ^132^Ba (0.10%), ^133^La production potential is much greater compared to ^132^La, illustrated in the difference between the absolute and isotopically weighted cross-sections shown in Figs. 2 and 3, respectively.

To compare ^133/135^La to ^132/135^La in this study, irradiations were performed with a target incident beam energy of 11.9 MeV. Figure 2 suggests irradiations at 11.9 MeV would result in the production of ^135^La and ^132^La via the ^135^Ba(p,n)^135^La, ^136^Ba(p,2n)^135^La, and ^132^Ba(p,n)^132^La reactions, while just avoiding the start of ^133^La production via the ^134^La(p,2n)^133^La reaction, as also described by Aluicio-Sarduy et al.^5^.

Other prominent cross sections at either 22 MeV or 11.9 MeV that are not depicted in Figs. 2 and 3 suggest unavoidable production of short-lived ^130^La (t_1/2_ = 8.7 min) via the ^130^Ba(p,n)^130^La reaction, ^131^La (t_1/2_ = 59.2 min) via the ^132^Ba(p,2n)^131^La reaction, ^134^La (t_1/2_ = 6.45 min) via the ^134^Ba(p,n)^134^La and ^135^Ba(p,2n)^134^La reactions, and ^136^La (t_1/2_ = 9.87 min) via the ^136^Ba(p,n)^136^La and ^137^Ba(p,2n)^136^La reactions. Significant cross sections are also present for the long-lived ^137^La (t_1/2_ = 6.2·10^4^ y) via the ^137^Ba(p,n)^137^La and ^138^Ba(p,2n)^137^La reactions, and ^138^La (t_1/2_ = 1.03·10^11^ y) via the ^138^Ba(p,n)^138^La reaction.

### Automated separation of ^133/135^La

^133/135^La was separated using modified aspects of a method described by Aluicio-Sarudy et al.^5^. The reactor vessel within its shield was transferred into the lead castle, the sealed target was opened by peeling back the Al cover, and a suction line was attached. The reactor vessel was filled with 10 mL of 18 MΩ·cm water, dissolving the ^nat^Ba target material in 5 min. The Ag target disc was removed, and 10 mL of 6 N HNO_3_ was added to the reactor to bring the overall concentration to 3 N HNO_3_. 3 N HNO_3_ was selected to reduce possible degradative effects of concentrated 6 N HNO_3_ on the branched DGA resin. The target solution was withdrawn from the reactor and passed through two Acrodisc 32 mm diameter filters with 5 µm Supor membranes in parallel to capture any solid material such as ^nat^Ba salts and oxides resulting from the dissolution stage. Following filtration, the target solution was passed through a SPE cartridge containing 0.25 g of branched DGA resin, and washed with 50 mL of 3 N HNO_3_ to remove residual Ba and other metal impurities, followed by 5 mL of 0.5 N HNO_3_. [^133/135^La]LaCl_3_ was eluted using 1 mL of 0.1 N HCl. Following a decay period of 5 days (to permit the decay of the short-lived ^107^Cd and longer-lived ^106m^Ag) the Ag disc was removed and cleaned in reagent grade 10 N HCl for reuse. For the comparative aspects, ^132/135^La was separated using the same process.

### Activity measurement and radionuclidic purity analysis

After separating the [^133/135^La]LaCl_3_ product, its radionuclidic purity was determined by gamma-ray spectroscopy using a high purity germanium (HPGe) detector. Calibrations for efficiency and energy were performed using NIST traceable Eckert & Ziegler Isotope Products Inc. γ-ray sources. Activities of La isotopes of interest were quantified using the efficiency-corrected HPGe measurements.

### Elemental purity analysis

Inductively-coupled plasma optical emission spectrometry (ICP-OES) analysis was performed to quantify elemental impurities in the [^133/135^La]LaCl_3_ samples after allowing 10 days for residual ^135^La to decay. The amounts of Zn, Fe, Al, Ba, Ag, In, Sn, and Cu were determined for each sample using calibrations obtained by measuring dilutions of elemental standards of known concentrations.

### Radiolabeling of DOTA and macropa with ^133/135^La

Following processing on the NEPTIS synthesis unit, the ^133/135^La radionuclide was eluted in 1 mL of 0.1 N HCl. 500 µL of [^133/135^La]LaCl_3_ was withdrawn, and the activity was measured. This solution was diluted with 50 µL of NaOAc buffer (pH 9.0) to adjust to pH 4.5. 100 µL of the ^133/135^La solution was reacted with 0.5 µg, 5 µg, and 20 µg of DOTA and macropa dissolved in 50 µL of 18 MΩ·cm water, at 80 °C for 30 min for DOTA and room temperature (22 °C) for 10 min for macropa. Each reaction solution was analyzed using radio-TLC on silica plates to determine radiochemical purity and incorporation with 0.1 M citric acid buffer as the mobile phase, with the R_f_ of free ^133/135^La = 0.9–1.0, [^133/135^La]La-DOTA = 0.1–0.2, and [^133/135^La]La-macropa = 0–0.1.

## Results

### Cyclotron targetry

Prior to longer irradiations, initial tests were performed with ^nat^Ba targets at beam currents ranging from 1–20 µA to investigate target properties and durability. After irradiation and automated separation, HPGe analysis was performed on the Ag targets. For 11.9 MeV runs, analysis indicated small activities of ^107^Cd (t_1/2_ = 6.5 h) and ^109^Cd (t_1/2_ = 461.4 d) were produced via the ^107^Ag(p,n)^107^Cd and ^109^Ag(p,n)^109^Cd reactions. For 22 MeV runs, following the 3-h decay period, analysis indicated small activities of ^107^Cd, ^109^Cd, and ^106m^Ag (t_1/2_ = 8.28 d). For both beam energies in this study, the targets did not activate significantly, and the majority of the activity present was ^107^Cd and ^106m^Ag, which decayed significantly after several days. Following a 5-day decay period the targets were deemed acceptable for handling and reuse after placing the target in 10 N HCl to clean its surface. For all irradiations, none of the sealed Ag targets showed signs of physical degradation, with multiple target discs being reused upwards of 10 times.

### ^133/135^La isotope production

Average activities (n = 3) of La isotopes of interest at 11.9 MeV and 22 MeV are given as a function of time after EOB in Table 1, and several ratios of La isotopes of interest are given as a function of time after EOB in Table 2.

**Table 1:** Average activities of La isotopes of interest at time-points after EOB for 500 µA·min runs (n = 3) at 22 MeV and 11.9 MeV proton beam energies.

Time after EOB (h)	22 MeV irradiation					11.9 MeV irradiation		
	Activity of La isotopes (MBq)					Activity of La isotopes (MBq)		
	^135^La	^133^La	^132^La	^131^La	^134^La + ^136^La	^135^La	^132^La	^134^La + ^136^La
0	166	231	0.38	19	5105	17.9	0.82	1394
1	160	193	0.33	9	60	17.3	0.71	15
2	155	162	0.29	4.7	0.86	16.7	0.62	0.22
3	149	136	0.25	2.3	0.013	16.1	0.53	0.00
4	144	114	0.21	1.1	0.00	15.6	0.46	0.00
6	134	80	0.16	0.28	0.00	14.5	0.35	0.00
8	125	56	0.12	0.07	0.00	13.5	0.26	0.00
12	108	28	0.068	0.00	0.00	11.7	0.15	0.00
24	71	3.3	0.01	0.00	0.00	7.7	0.03	0.00
48	30	0.0	0.00	0.00	0.00	3.3	0.00	0.00

**Table 2:** Activity ratios of La isotopes of interest at time-points after EOB for 500 µA·min runs (n = 3) at 22 MeV and 11.9 MeV proton beam energies.

Time after EOB (h)	22 MeV irradiation	22 MeV irradiation	11.9 MeV irradiation
	Activity ratio of ^135^La to ^133^La	Activity ratio of ^133^La to ^132^La	Activity ratio of ^135^La to ^132^La
0	0.72	608	18
1	0.83	588	20
2	0.95	569	22
3	1.1	550	24
4	1.3	532	27
6	1.7	498	34
8	2.2	465	42
12	3.9	407	64
24	22	273	236
48	645	122	3172

At 22 MeV, 500 µA·min runs (n = 3) yielded 231 ± 8 MBq ^133^La, and 166 ± 5 MBq ^135^La. Saturated yields were 161 ± 5.5 MBq/µA for ^133^La, and 561 ± 17 MBq/µA for ^135^La. Significant amounts of ^134^La and ^136^La were present at EOB (1191 ± 96 MBq and 3914 ± 384 MBq, respectively), however owing to their short half-lives (6.45 min and 9.87 min, respectively), they decayed to negligible levels after 3-h post-EOB. Short-lived ^130^La (8.7 min half-life) was observed and undetectable after the 3-h decay period. ^132^La was produced (0.38 ± 0.03 MBq at EOB), indicating its production reactions were largely bypassed. Co-production of ^131^La was observed (19.0 ± 1.2 MBq at EOB), however owing to its relatively short half-life (59.2 min), it decayed significantly during the 3-h decay period. TENDL 2019 cross sections indicated production of long-lived ^137^La and ^138^La, however, this was not quantified due to their extremely long half-lives.

For the comparison ^132/135^La production runs at 11.9 MeV, 500 µA·min runs (n = 3) yielded 0.82 ± 0.06 MBq ^132^La and 17.9 ± 0.8 MBq ^135^La at EOB. Saturated yields were 0.70 ± 0.03 MBq/µA for ^132^La, and 60.6 ± 2.8 MBq/µA for ^135^La. Significant amounts of ^134^La and ^136^La were also observed at EOB (411 ± 37 MBq and 2462 ± 94 MBq, respectively), which decayed to undetectable levels after the 3-h decay period. Cross-sections generated by TENDL 2019 indicated the production of long-lived ^137^La and ^138^La. However, production was also not quantified owing to their long half-lives.

As shown in Table 2, the activity ratio of ^135^La to ^133^La at 22 MeV is much lower than the ratio of ^135^La to ^132^La at 11.9 MeV, resulting in a much greater PET imaging potential for a given total activity. At 22 MeV, the activity ratio of ^133^La to ^132^La remains large throughout the time intervals, suggesting that the production of the ^132^La impurity was minimized.

### Automated separation of ^133/135^La

To determine dissolution time, several Ba targets were dissolved in the reactor with 10 mL of water, with the time required to completely dissolve the target ranging from 4 to 5 min. A dissolution time of 5 min was selected for production run separations to provide a sufficient time margin. The DGA resin was preconditioned with 3 N HNO_3_ so the NEPTIS unit was prepared to receive the activity. The final product elution in 1 mL of 0.1 N HCl was calibrated to capture the maximum ^133/135^La activity while avoiding excess dilution of the solution.

From the start of NEPTIS separation to the completion of product elution took ~ 35 min. Over 88% of decay-corrected ^133/135^La activity was consistently recovered from the automated synthesis. Residual decay activities were 3% of the total in the branched DGA resin, 3% in the dissolution reactor, 2% in the two reactor filters, with the remainder (≤ 4%) in the waste.

### Radionuclidic and elemental purity analysis

For irradiations at 22 MeV beam energies, small amounts of ^131^La and ^132^La were detected by HPGe gamma-ray spectroscopy performed on the ^133/135^La eluate product after NEPTIS separation and a 3-h decay period. For 500 µA·min runs (n = 3) at 22 MeV, the ^131^La and ^132^La activities back-calculated to EOB were 19 ± 1.2 MBq and 0.38 ± 0.03 MBq, respectively.

The decay of ^133^La resulted in small activities of its daughter nucleus ^133^Ba (t_1/2_ = 10.6 y). However, the resulting activity of ^133^Ba after the complete decay of ^133^La was approximately three orders of magnitude lower than the IAEA 1 MBq consignment level exemption limits^13^. No other radionuclidic impurities were observed in the ^133/135^La product.

After allowing the ^133/135^La eluate to decay for 10 days, an ICP-OES analysis was performed to investigate trace metal contaminants against a known mixture standard containing Zn, Fe, Al, Ba, Ag, In, Sn, and Cu. Metal impurities (n = 3 runs) are presented in Table 3.

**Table 3:** Comparative ICP-OES elemental contaminant analysis of the [^133/135^La]LaCl_3_ product.

Metal	Concentration (ppb)
Zn	76 ± 55
Fe	16.8 ± 11.7
Al	37 ± 19
Ba	1150 ± 360
Ag	1.9 ± 0.3
In	3.1 ± 0.9
Sn	126 ± 104
Cu	5.3 ± 0.4

### Radiolabeling of DOTA and macropa chelators with ^133/135^La

Table 4 summarizes the experimental results of ^133/135^La radiolabeling with DOTA and macropa chelators. Radiolabeling with the tetraaza-macrocyclic chelator DOTA was performed with ^133/135^La at 80 ºC for 30 min and analyzed with radio-TLC. The [^133/135^La]La-DOTA complex remained close to the TLC baseline (R_f_ = 0.1–0.2) while the unreacted ^133/135^La migrated toward the solvent front (R_f_ = 0.9–1.0). The incorporation (n = 3) of ^133/135^La for DOTA labeling was 99.1 ± 0.6%, 98.8 ± 0.5%, and 97.9 ± 1.2% for 20, 5, and 0.5 µg, respectively. Complete labeling of DOTA with ^133/135^La was achieved up to 1.2 nmol of DOTA, with a corresponding apparent ^135^La molar activity (n = 3) of 47 ± 9 GBq/µmol and ^133^La molar activity (n = 3) of 33 ± 5 GBq/µmol .

**Table 4.** ^133/135^La radiolabeling results with DOTA and macropa chelators.

Chelator mass (µg)	[^133/135^La]La-DOTA incorporation (%)	[^133/135^La]La-macropa incorporation (%)
20	99.1	99.3
5	98.8	99.5
0.5	97.9	98.1

Radiolabeling with the eighteen-membered macrocyclic chelator macropa was performed with ^133/135^La at room temperature (22 ºC) for 10 min, and analyzed with radio-TLC.

The [^133/135^La]La-macropa complex remained at the TLC baseline (R_f_ = 0–0.1) while the unreacted ^133/135^La migrated toward the solvent front (R_f_ = 0.9–1.0). The incorporation (n = 3) of ^133/135^La for macropa labeling was 99.3 ± 0.5%, 99.5 ± 0.7%, and 98.1 ± 1.1% for 20, 5, and 0.5 µg, respectively. Complete labeling of macropa with ^133/135^La was achieved up to 0.85 nmol of macropa, with a corresponding apparent ^135^La molar activity (n = 3) of 44 ± 8 GBq/µmol and ^133^La molar activity (n = 3) of 30 ± 4 GBq/µmol.

## Discussion

This study presents a high-yield cyclotron production avenue for a novel ^133/135^La theranostic pair using a new sealed target design. Automated separation and purification produced a chemically pure product, with radiochemistry validating the feasibility of the ^133/135^La theranostic pair using several common radiometal chelators.

Table 5 outlines the positron decay characteristics and notable gamma rays for ^133^La, ^132^La, and several other common isotopes used for PET. ^132^La has a higher positron branching ratio (41.2%) compared to ^133^La (7.2%), producing more 511 keV emissions for a given sample activity. Initially, this higher branching ratio would seem advantageous for PET imaging. However, positrons emitted by ^132^La have a much higher 1.29 MeV average and 3.67 MeV maximum energy compared to ^133^La positron emissions, which have a low, more desirable 0.463 MeV average and 1.02 MeV maximum positron energy. Since higher positron energies are correlated with lower PET imaging spatial resolution^14,15^, this implies that ^133^La would have superior PET imaging quality compared to ^132^La.

**Table 5:** Positron decay characteristics and notable gamma rays for ^133^La, ^132^La, and other common PET isotopes^9^.

Isotope	Half-life	Positron branching ratio (%)	Mean positron energy (MeV)	Maximum positron energy (MeV)	Gamma ray energy and intensity
^133^La	3.91 h	7.2	0.461	1.02	279 keV (2.4%), 302 keV (1.6%), 291 keV (1.4%), 846 keV (0.4%), 1099 keV (0.2%)
^132^La	4.82 h	41.2	1.29	3.67	465 keV (76%), 567 keV (15.7%), 1910 keV (9%), 663 keV (9%), 1032 keV (7.8%), 540 keV (7.7%)
^18^F	110 min	96.7	0.250	0.634	None
^68^ Ga	67.7 min	88.9	0.829	1.90	1077 keV (3.2%), 1883 keV (0.14%), 1261 keV (0.1%)
^64^Cu	12.7 h	17.6	0.278	0.653	1345 keV (0.48%)
^44^Sc	3.97 h	94.3	0.632	1.47	1157 keV (99.9%), 1499 keV (0.91%), 2656 keV (0.11%)
^89^Zr	78.4 h	22.7	0.396	0.902	909 keV (99%), 1713 keV (0.75%), 1744 keV (0.12%)
^11^C	20.4 min	99.8	0.386	0.960	None
^82^Rb	1.26 min	95.4	1.48	3.38	777 keV (15.1%), 1395 keV (0.53%), 698 keV (0.15%), 1475 keV (0.09%)

The potential for improved PET scanning resolution of ^133^La over ^132^La could permit more accurate imaging to track the treatment of small tumors and metastases, complementing high LET targeted radionuclide therapy such as alpha particle or AET, which are both well suited for eradicating small metastatic tumors.

As shown in Table 5, ^132^La has high energy gammas with a significant abundance, whereas ^133^La has lower energy gammas with a much lower abundance. ^132^La has a maximum gamma energy of 1909.91 keV at 9% abundance, whereas ^133^La has a maximum gamma energy of 1099 keV with a 0.2% abundance. The lower energy and much lower abundance of the ^133^La gamma rays should simplify handling and reduce the dose to patients upon injection for equivalent imaging activities, even though a greater activity of ^133^La might be required due to the lower positron branching ratio of ^133^La. In addition to potentially reducing the patient dose, the gamma ray energy distribution of ^133^La could improve PET scanner imaging spatial resolution.

The ^132^La 465 keV (76%) and 567 keV (14.7%) high abundance gamma rays are within a typical 350–650 keV PET scanner energy window used to detect the 511 keV annihilation gamma rays^15^, which could lead to excess spurious coincidences within the scanner timing window, and interfere with image quality. ^133^La has no gamma rays with energies within a typical PET scanner energy window, which should result in no spurious coincidences. Additionally, as previously depicted in Table 2, the much lower activity ratio of ^135^La to ^133^La produced at 22 MeV, compared to the ratio of ^135^La to ^132^La produced at 11.9 MeV, should significantly reduce the relative amount of spurious coincidences in the PET scanner energy window from the ^135^La 480.5 keV gamma ray.

Comparing ^133^La to other PET isotopes in Table 5 shows that its respective mean and maximum positron energies of 0.461 MeV and 1.02 MeV are higher than those of ^64^Cu and ^18^F, comparable to those of ^11^C, and ^89^Zr, and lower than those of ^132^La, ^68^Ga, ^44^Sc, and ^82^Rb.

The ubiquitous ^18^F has a very low positron energy that provides a sharp image, and ^11^C has a similar positron energy to ^133^La. However, the shorter half-lives of ^18^F and ^11^C limit investigating longer biological processes. ^64^Cu has low energy positron emissions, a longer half-life, and β^–^ emissions that enable theranostics, however cyclotron production requires expensive isotopically enriched target material due to the low 0.009% natural abundance of ^64^Ni. ^89^Zr has the longest half-life of the listed isotopes, permitting users to examine longer biological processes, however, it has several high energy gamma rays (909 keV (99%), 1713 keV (0.75%), and 1744 keV (0.12%)), which greatly increase the patient dose and shielding requirements.

^68^Ga has become a widely used radiometal for PET owing to its high positron branching ratio, sufficient half-life, and demonstrated chemistry. ^68^Ga is easily accessible via ^68^Ge/^68^Ga generators, and alternative cyclotron production routes have demonstrated potential to further enhance ^68^Ga supply^10^. However, its higher positron energies compared to ^133^La, ^18^F, and ^64^Cu result in lower imaging spatial resolution^16^, and it also has several high energy gamma rays, notably 1077 keV (3.2%), that increase shielding requirements. Despite having a longer half-life than ^68^Ga, the higher energy positrons of ^44^Sc compared to ^133^La, ^18^F, and ^64^Cu would also result in a lower image resolution while complicating handling and contributing significantly to patient dose with its 1157 keV (99.9%) gamma-ray emissions.

^132^La has a similar half-life to ^133^La. However, it has drawbacks including high positron emission energies and high energy and abundance gamma emissions. ^82^Rb also has high energy positrons, though this is acceptable given its role in imaging large cardiac structures.

From the previous comparisons, the relatively low positron energies, gamma energies, and gamma abundances of ^133^La imply higher imaging resolution than ^132^La, ^68^Ga, ^44^Sc, and a comparable imaging resolution to ^11^C and ^89^Zr. ^133^La appears to be an attractive radiometal candidate for PET applications requiring a high scanning resolution, with its relatively long isotopic half-life, ease of handling, and low patient dose. Quantifying ^133^La dosimetry in future studies is worth pursuing.

Significant advantages arise from our production method and the intrinsic properties of the ^133/135^La pair, compared to the currently produced ^132/135^La pair. Our production technique using a 24 MeV cyclotron with a new sealed target design allows high yield on-demand production.

Without an effective sealed target design, the metallic ^nat^Ba ejects BaO dust into its surroundings as it rapidly oxidizes in the atmosphere, posing a potential radioactive contamination hazard during irradiation and target retrieval. Our sealed target design eliminates this issue through the secure encapsulation of the sensitive ^nat^Ba target material with a durable bond between the Al target cover, In wire, and Ag disc. Furthermore, the sealed solid target design production method is robust and efficient, and the completed targets are easy to store and handle pre- and post-irradiation.

Irradiated Ag targets became activated with significant activity of ^107^Cd, and small activities of ^109^Cd, and ^106m^Ag. Despite the 8.28-day half-life of ^106m^Ag, after allowing for a several day decay period, residual activity in Ag targets was low enough for target reuse.

Cyclotron irradiations at 22 MeV achieved high-yield production of ^133^La and ^135^La, while only producing extremely small activities of ^132^La relative to ^133^La. Even though there was an appreciable drop in beam energy across the 0.8 mm ^nat^Ba pellet (22 MeV to 18.3 MeV calculated by SRIM), this did not result in any significant increase in ^132^La production since the ^132^Ba(p,n)^132^La cross-section remains low across this energy range, and ^132^Ba has a low isotopic abundance of 0.10%. Avoiding the onset of the higher energy ^134^Ba(p,3n)^132^La reaction was important since the 2.42% isotopic abundance of ^134^Ba would produce a much greater activity of the ^132^La impurity compared to the ^132^Ba(p,n)^132^La reaction. Minimal production of ^131^La via the ^132^La(p,2n)^131^La reaction was observed, with any activity produced significantly decaying during the 3-h post-EOB decay period, due to its 59.2 min half-life. To further reduce radionuclidic impurities, removing the 0.1% of ^132^Ba natural abundance via isotopic enrichment of ^nat^Ba should allow the near-complete removal of ^132^La production from the ^132^Ba(p,n)^132^La reaction and remove ^131^La from the ^132^La(p,2n)^131^La reaction, leaving only ^133^La and ^135^La after the 3-h decay period. This enriched target material would also enable cyclotrons with an energy lower than 22 MeV to produce radionuclidically pure ^133/135^La (although at lower production yields). Other isotopic enrichments could potentially increase production yields of ^133^La or ^135^La. However, the additional cost and availability of enriched Ba target material, as opposed to using relatively inexpensive ^nat^Ba, would be an important factor to evaluate.

The decay of ^133^La forms the daughter ^133^Ba (t_1/2_ = 10.6 y), which decays to form stable ^133^Cs. However, ^133^Ba activity resulting from the decay of its ^133^La is comparatively far smaller, and approximately three orders of magnitude below IAEA consignment exemption quantities^13^. Any additional dose from a ^133^La PET scan resulting from the very small amount of the ^133^Ba daughter would be minimal due to its extremely low activity resulting from its far longer half-life relative to ^133^La, low maximum gamma energy of 383 keV, and rapid excretion from the body^17,18^. A study by Newton et al.^18^ injected 72.4–79.5 kBq ^133^Ba into the bloodstream of healthy human volunteers and studied the full-body retention of ^133^Ba up to 13 y after injection. The majority of injected ^133^Ba was rapidly cleared from the body (74–90% within 10 d), with residual activity continuously excreted as time progressed.

Additionally, depending on the properties of the targeting vector used to deliver ^133^La, some of the ^133^La injected for a PET scan could be excreted before decaying to ^133^Ba, owing to its 3.92 h half-life. Therefore, pharmacokinetic studies would be useful to assess the in vivo distribution of ^133^La radiopharmaceuticals and its ^133^Ba decay daughter. As considered with cyclotron produced ^99m^Tc, it would be useful to do a future evaluation on the significance of long-lived impurities and decay products on the patient dose^19^.

The automated separation of ^133/135^La from the ^nat^Ba target material using a NEPTIS unit achieved a decay corrected activity recovery of 88% while producing a highly pure product ready for radiolabeling. In the future, ^133/135^La radiolabeling and radiopharmaceutical syntheses can be added to the automated synthesis process to create a final product for research or clinical use.

Radiolabeling of DOTA and macropa was successful, with high incorporations observed with each chelator. Concerning chemistry, the production of significant amounts of the “stable” isotopes ^138^La and ^137^La, could provide competition to ^133/135^La or ^132/135^La during radiolabeling, since their reaction cross sections are much larger than those of ^133/135^La at 22 MeV and ^132/135^La at 11.9 MeV. However, TENDL 2019 reaction cross-sections for the ^138^Ba(p,n)^138^La, ^137^Ba(p,n)^137^La, and ^138^Ba(p,2n)^137^La reactions indicate the amount of ^137/138^La relative to ^133/135^La produced at 22 MeV is smaller than that of ^137/138^La relative to ^132/135^La produced at 11.9 MeV^12^. This implies that irradiating ^nat^Ba at 22 MeV could be advantageous over 11.9 MeV from a chemistry perspective, with a lower proportion of “stable” ^137/138^La isotopes competing during radiolabeling.

^133/135^La has potential as a theranostic pair for PET imaging and AET in targeted radionuclide therapy. With 11 Auger electrons per decay, ^135^La produces a significant amount of high LET radiation, which is especially suited for killing metastases. With an appropriate targeting vector, ^133^La could be used to image and ^135^La to kill tumor cells.

Existing low current 11.9 MeV cyclotron ^132/135^La production requires several-hours of long irradiations to produce small activities for limited pre-clinical applications. In contrast, much higher cross-sections for ^133/135^La at 22 MeV allow a significantly shorter irradiation time producing over an order of magnitude more ^133/135^La compared to ^132/135^La, and significantly, large amounts of ^133^La relative to ^135^La as previously depicted in Table 2. This large-scale ^133^La production compensates for the lower positron branching ratio of ^133^La compared to ^132^La. Additionally, compared to the small ^132^La/^135^La ratio shortly after EOB, the far larger ^133^La/^135^La ratio allows more flexibility with imaging and therapy.

There is a significant potential increase in PET imaging when using the ^133/135^La product soon after the 3-h decay period post-EOB, as well as allowing large amounts of pure Auger therapy with a longer decay period after EOB.

A typical ^18^F activity of 300–400 MBq is used for clinical PET imaging^20^, and a typical ^68^ Ga activity of 1.59 MBq/kg is suggested^21^. It would be a challenge to produce a ^132^La activity equivalent to a typical ^18^F or ^68^ Ga dose with current ^132/135^La production methods unless isotopically enriched Ba target material was used. In contrast, it should be far easier to reach a clinically relevant ^133/135^La activity with a 22 MeV irradiation of a ^nat^Ba target. The much greater yield of ^133/135^La with our 22 MeV higher energy production method should enable clinically relevant amounts of activity to be produced with relatively short irradiations.

It should be noted that not all PET centers have access to a cyclotron that can reach 22 MeV, so ^133/135^La production will be limited to those centers with sufficiently high beam energy. However, the relatively long half-lives of ^133^La (3.9 h) and ^135^La (19.5 h) would permit regional distribution of the ^133/135^La theranostic pair.

## Conclusion

We have developed a high yield and cost-effective method of producing a novel theranostic pair, ^133/135^La. Our production technique uses a new type of sealed solid target that is robust, simple to manufacture, significantly improves target handling, and contains reusable components. Production yields of ^133/135^La at 22 MeV are over an order of magnitude higher than existing ^132/135^La production techniques, enabling clinically relevant ^133/135^La activites to be produced at low cyclotron beam currents and relatively short irradiation times, without expensive isotopically enriched Ba target material. ^133/135^La shows intriguing imaging potential due to its much lower positron energy and far lower gamma-ray energies and abundances compared to ^132/135^La, with potential applications for treating cancer metastases as a PET/AET theranostic pair. Accordingly, ^133/135^La appears to be an attractive radiometal theranostic candidate for PET applications requiring high scanning resolution, a relatively long half-life, ease of handling, and lower patient dose. This study demonstrated the potential for high-yield ^133/135^La production via ^nat^Ba irradiation at sites with a medical cyclotron that can reach 22 MeV, meeting increasing demands for pre-clinical and potential clinical applications for ^133/135^La radiopharmaceuticals.
